# Disentangling food addiction–related symptom profiles in anorexia nervosa: a latent class analysis and clinical implications

**DOI:** 10.1186/s40337-026-01611-z

**Published:** 2026-04-19

**Authors:** Matteo Aloi, Marianna Rania, Elvira Anna Carbone, Renato de Filippis, Ettore D’Onofrio, Lavinia Rotella, Daria Quirino, Antonino Carcione, Cristina Segura-Garcia

**Affiliations:** 1 Department of Clinical and Experimental Medicine, University of Messina, Via Consolare Valeria, Messina, 98125 Italy; 2 Outpatient Unit for Clinical Research and Treatment of Eating Disorders, University Hospital “Renato Dulbecco”, Catanzaro, Italy; 3 Department of Health Sciences, University “Magna Graecia” of Catanzaro, Catanzaro, Italy; 4 Department of Medical and Surgical Sciences, University “Magna Graecia” of Catanzaro, Catanzaro, Italy; 5 Third Centre of Cognitive Psychotherapy, Italian School of Cognitive Psychotherapy (SICC), Rome, Italy; 6 Department of Human Science, University “Guglielmo Marconi”, Rome, Italy

**Keywords:** Food addiction, Anorexia nervosa, Eating disorders, Person-approach, Treatment

## Abstract

**Background:**

The construct of Food Addiction (FA) has generated growing interest in the context of Anorexia Nervosa (AN), especially for its potential clinical implications. While AN is traditionally associated with restrictive eating patterns, recent findings suggest that FA symptoms may also be present in this population, complicating the clinical picture. This study aimed to explore FA symptomatology in individuals with AN using a person-centered approach and to identify psychological variables associated with different FA profiles.

**Methods:**

A sample of 202 individuals with a diagnosis of AN (including AN-R, AN-BP, and Atypical AN) completed the YFAS 2.0. A Latent Class Analysis (LCA) was performed to identify subgroups based on FA symptoms. Chi-square tests assessed associations between latent classes and AN subtypes. Binary logistic regressions explored psychological predictors of class membership, including eating psychopathology (EDE-Q), depressive symptoms (BDI-II), emotion dysregulation (DERS), and metacognitive abilities (MSAS).

**Results:**

LCA identified three latent classes: Class 1 (Addicted, 11.4%), characterized by high endorsement of FA symptoms; Class 2 (High-Risk, 33.7%), displaying moderate FA symptom severity; and Class 3 (Non-Addicted, 54.9%), showing minimal FA symptomatology. No significant association emerged between class membership and AN diagnostic subtype (χ²(4) = 3.085, *p*=.544). Logistic regressions revealed that impulsivity significantly predicted membership in the Addicted class (OR = 4.119, *p*<.05), while membership in the Non-Addicted class was associated with lower eating psychopathology, better metacognitive skills, and reduced impulsivity. Belonging to the High-Risk class was predicted by higher eating concern (OR = 2.618, *p*<.05), lower metacognition (OR=0.566, *p*<.05) and greater difficulties with emotional awareness (OR = 1.62; *p*<.01).

**Conclusions:**

These findings highlight the presence of distinct FA profiles within AN, independent of traditional diagnostic categories. The Addicted and High-Risk classes showed greater psychopathology and dysregulation. Notably, the High-Risk group also exhibited impaired metacognition, suggesting potential vulnerability despite moderate symptom expression. In contrast, the Non-Addicted class showed a more adaptive profile. LCA may support the development of personalized treatment strategies by targeting FA-related features, including interventions to improve emotion regulation and metacognitive functioning.

## Abstract (plain language summary)

This study explored whether people with anorexia nervosa (AN) can also show signs of “food addiction,” a pattern of feeling driven to eat in an addictive way—such as craving food, feeling out of control, or experiencing distress about eating. Although AN is usually thought of as a disorder of food restriction, recent evidence shows that some people with AN may still struggle with thoughts and behaviours similar to addiction. We studied over 200 people with different types of AN and found three groups. One group clearly showed food addiction, another group showed some warning signs (“high-risk”), and the third group did not show signs of food addiction. These groups were not linked to a specific AN subtype, meaning that addictive-like eating thoughts can appear in any form of AN, even when there is no obvious binge eating. People in the food-addicted or high-risk groups had more severe eating disorder symptoms, more difficulties managing their emotions, and more problems with recognising their own and others’ thoughts and feelings (a skill called metacognition). Our findings suggest that helping people with AN strengthen these emotional and thinking skills may reduce the risk of food addiction and support recovery.

## Background

Anorexia nervosa (AN) is a severe psychiatric disorder featured by excessive preoccupation with not gaining weight, persistent restriction of energy intake, and disturbance of body image [1]. Although it has been traditionally conceptualized as a restrictive disorder, increasing evidence implies that the clinical presentation of AN is very heterogeneous and some subjects present behavioral characteristics in common with those seen in addictive disorders [2, 3]. This complexity has fueled growing interest in the concept of food addiction (FA), which refers to an addictive-like pattern of eating behavior marked by loss of control, craving, withdrawal, and continued use despite negative consequences [4, 5]. Although FA has been primarily investigated in obesity and binge-eating disorder (BED) [6–8], more recent investigations have also found that it occurs in individuals with AN, which represents a conclusion of clinical and theoretical relevance [9, 10].

The co-occurrence of FA with AN may appear to be a contradiction since AN has historically been associated with restraint, and not compulsive overeating. Nonetheless, several explanations have been posited. First, patients with AN, particularly the binge-purge subtype (AN-BP), may exhibit similar compulsive engagement with food-anchored thoughts and behaviors to addictive processes [11, 12]. Second, symptoms of FA may be a manifestation of hidden reward, impulse control, and metacognitive impairments, even in the absence of the overt presence of overeating [13]. Clinically, the identification of FA symptomatology in AN has the potential to uncover subgroups of patients with particular vulnerabilities and treatment needs, thereby enhancing diagnostic classification and individually tailored interventions.

The exploration of FA in AN is further complicated by its associations with transdiagnostic psychological dimensions. In general, high levels of eating disorder psychopathology have been linked to increased FA scores across all diagnostic groups [14, 15].

Depressive symptoms and impairment in emotion regulation also play a central role, given their established contribution to both addictive behaviors and eating disorders (EDs) [16–19]. Furthermore, metacognition impairments, defined as the ability to recognize one’s own and other individuals’ mental states [20] seem to be primary mechanisms for EDs [21–23]. In this vein, a recent latent profile analysis study in a large clinical sample supports the view that metacognition operates as a transdiagnostic factor across EDs, showing robust associations with symptom severity as well as with broader psychological and developmental correlates, including trauma histories and personality dysfunctions [24]. Impairments in metacognitive abilities like self-monitoring, differentiation/decentration and mastery may increase vulnerability to rigid thinking, inadequate self-regulation, and ineffective coping strategies, potentially amplifying FA symptoms in AN samples.

Despite these findings, little is known regarding how FA operates within AN and how it relates to these psychological variables. Prior research has largely adopted variable-centered approaches (e.g., correlations, regression), assuming homogeneity between people and potentially masking clinically important subgroups. Compared to person-centered methods such as latent class analysis (LCA), which allow for the description of distinctive profiles based on patterns of symptoms, a more differentiated portrayal of FA in AN is provided [25].

Identification of latent classes would indicate whether FA is a homogeneous concept within AN or whether subgroups with different degrees of eating psychopathology, depression severity, emotional dysregulation, and metacognitive functioning exist. To date, no study has previously investigated the FA construct with the LCA in the AN population.

According to the Yale Food Addiction Scale 2.0 (YFAS 2.0), FA is operationalized through 11 symptom domains reflecting substance use–like criteria, such as craving and preoccupation, loss of control, tolerance, withdrawal, and continued behavior despite negative consequences, along with clinically significant impairment or distress [26]. It is important to acknowledge that some of these symptoms, such as distress related to eating, avoidance of social situations involving food, or perceived negative consequences, partially overlap with core features of EDs, particularly AN. In AN, distress around eating and food-related avoidance often stem from weight- and shape-related fears rather than from compulsive overconsumption per se.

However, FA is not conceptualized here as a discrete diagnostic entity independent of EDs, but rather as a dimensional construct reflecting addictive-like processes that may cut across ED diagnoses. From this perspective, overlap with eating disorder psychopathology does not invalidate the construct, but instead highlights shared vulnerability processes. In individuals with AN, FA-related symptoms may capture additional mechanisms, such as compulsive engagement with food-related thoughts, heightened salience of food cues, perceived loss of control at the cognitive affective level, or impaired impulse control, which coexist with but are not reducible to restrictive eating pathology alone.

Consistent with this framework, all FA symptom domains defined by the YFAS 2.0, including impairment or distress, were retained in the present LCA. The aim was not to isolate pure addiction symptoms, but to examine how addiction-like features cluster within AN when FA is considered dimensionally. Excluding overlapping indicators would have artificially constrained the construct and reduced comparability with the broader FA literature, while treating impairment solely as an external validator would have limited the ability to distinguish profiles differing in functional severity.

The primary aim of this research was to identify distinct profiles of FA symptomatology in individuals with AN using a person-centered LCA approach.

The secondary aim was to examine whether the identified FA profiles differed in levels of ED psychopathology, depressive symptoms, emotion dysregulation, and metacognitive functioning.

Finally, we explored which psychological variables independently predicted membership in each FA profile, thereby clarifying the unique clinical features associated with each subgroup.

Based on previous research, we hypothesized that individuals with more severe FA symptoms would report greater ED psychopathology, more severe depression, greater emotion regulation difficulties, and impaired metacognitive functioning than individuals with fewer o no FA symptoms.

## Methods

### Participants

Participants were recruited from patients attending the Outpatient for Clinical Research and Treatment of Eating Disorders at the University Hospital “Renato Dulbecco” of Catanzaro (Italy) between July 2018 and July 2025, in the context of a cross-sectional observational study. Recruiting was consecutive on the first visit of the patients, after an elaborated explanation provided by the research team of the study’s aims and methods. The inclusion criteria required participants to be ≥ 14 years old, to meet DSM-5 diagnostic criteria for AN [1] and to provide their informed consent for their participation in the study. Exclusion criteria were as follows: presence of major psychiatric comorbidities (e.g., neurodevelopmental disorders, schizophrenia spectrum disorders, bipolar illness, or neurocognitive disorders), neurological or medical diseases (e.g., diabetes), current substance abuse or dependence within the last six months, and other medical illness or treatments that would likely affect eating behaviour.

Diagnostic assessment was conducted by trained psychiatrists using the Eating Disorder Examination (EDE 17.0D; [27]). Subsequently, participants completed self-report measures evaluating psychological constructs including depression, emotion dysregulation, and metacognition.

Of the 212 patients initially considered for participation, 10 were excluded during screening or enrolment. Specifically, five (2.3%) discontinued before completing the assessment, three (1.4%) met exclusion criteria due to intellectual disability, and two (1.0%) were deemed ineligible because of psychotic symptoms. Consequently, the final sample consisted of 202 patients [*N* = 135 Anorexia Nervosa - Restricting subtype (AN-R), *N* = 25 Anorexia Nervosa – Binge eating/Purging subtype (AN/BP), *N* = 42 Atypical AN] with a dropout rate of 4.7%.

Only those who agreed to take part in the study, provided signed informed consent, and proceeded to complete the full assessment protocol were included in the analyses. There were no missing data on either socio-demographic variables or assessment measures. The study was conducted in accordance with the ethical standards of the revised Declaration of Helsinki [28] and was approved by the Regione Calabria Sezione Area Centro Ethics Committee (protocol number: Prot. 66/15.03.2018). Written informed consent was obtained from all participants prior to questionnaire administration. In the case of minors, parents or legal guardians provided consent after being given a full explanation of the study.

### Measures

All participants completed the following battery of tests in a single administration time and in the same sequence:

- Yale Food Addiction Scale 2.0 The Italian version was administered to evaluate addiction-like eating behaviors over the previous 12 months. The instrument includes 35 items rated on an eight-point Likert scale (0 = never to 7 = every day), covering 11 symptom domains [26, 29]. Two scoring approaches are available. The first is a symptom count, reflecting the total number of criteria met (ranging from 0 to 11). The second provides a severity classification, consistent with DSM-5 substance use disorder thresholds: mild food addiction (FA; 2–3 symptoms), moderate FA (4–5 symptoms), and severe FA (≥ 6 symptoms). For a categorical FA diagnosis (present/absent), participants must endorse at least two criteria and report significant impairment or distress. In accordance with the YFAS 2.0 scoring protocol, symptom endorsement was determined using predefined frequency thresholds rather than raw item scores. Each of the 11 food addiction symptoms is assessed by multiple items, and a symptom was coded as “present” only when participants’ responses met or exceeded the validated cut-off criteria specified in the original validation work [26]. In this study, the YFAS 2.0 demonstrated good internal consistency, with a Kuder–Richardson reliability coefficient of 0.85.
- Beck Depression Inventory-II (BDI-II): This scale assesses depressive symptom severity across 21 items [30]. Items are rated on a 4-point Likert scale ranging from 0 to 3, with higher scores indicating greater depressive symptom severity. Standard cut-off ranges are as follows: 0–13 = minimal depression, 14–19 = mild depression, 20–28 = moderate depression, and 29–63 = severe depression. In the present study, internal consistency was excellent, with McDonald’s ω = 0.90.
- Eating Disorder Examination (EDE): The EDE is a semi-structured interview designed to assess the severity of eating disorder psychopathology in the last three months [27]. It includes four subscales: Restraint, Eating Concern, Weight Concern, and Shape Concern, that together yield a global score. Items are rated by the interviewer on a 7-point scale (0–6), with higher scores indicating greater severity of eating disorder psychopathology. Further, it evaluates behavioural signs of EDs like binge eating, vomiting, diuretic and laxative use, excessive physical activity, and food restriction. In the present study, internal consistency (McDonald’s ω) was 0.77 for Restraint, 0.76 for Eating Concern, 0.77 for Weight Concern, 0.82 for Shape Concern, and 0.85 for the Global score.
- Metacognition Self-Assessment Scale (MSAS): The MSAS is an 18-item self-report tool designed to assess metacognitive functioning [31]. Responses are rated on a Likert-type scale ranging from 1 (never) to 5 (always), with lower scores reflecting greater impairment in self-appraisal of metacognitive abilities. The scale assesses four metacognitive sub-functions: self-monitoring, differentiation/decentration, mastery, and the capacity to understand others’ minds. In the present study, internal consistency (McDonald’s ω) ranged from 0.83 for Mastery to 0.92 for Self-monitoring.
- Difficulties in Emotion Regulation Scale (DERS): The 36-item DERS measures emotion dysregulation across six subscales using a 5-point Likert scale ranging from 1 (almost neve”) to 5 (almost always) [32]: (a) nonacceptance of emotions, (b) difficulties pursuing goals under strong emotions, (c) difficulties controlling impulses when distressed, (d) lack of emotional awareness, (e) limited access to effective regulation strategies, and (f) lack of emotional clarity. Subscale scores can be summed to yield a total score, with higher scores indicating greater difficulties in emotion regulation. In this sample, McDonald’s ω ranged from 0.75 (Goals) to 0.88 (Awareness).

### Statistical analysis

LCA was performed with the package poLCA in R [33], which maximizes class models using maximum likelihood. To reduce problems of non-convergence and local solutions, analyses were run with repeated random starting values (*n* = 10) and high iterations (*n* = 5000), retaining the solution with the optimal fit. LCA was performed sequentially, beginning with a one-class model and adding classes successively. The process yields class membership probabilities as well as item-response probabilities conditional on class membership that are used for interpretation of the final model. Statistical fit indices, parsimony, and class interpretability guided model selection. Indices reported were log-likelihood, Akaike Information Criterion (AIC), Bayesian Information Criterion (BIC), and the likelihood ratio/deviance statistic (G^2) [25], with lower values reflecting better fit. We evaluated classification accuracy using entropy (range 0–1), with values above 0.80 interpreted as evidence of adequate class separation [34].

While there are no formally agreed rules for minimum sample size in LCA, earlier evidence indicates that adequacy is related to the number of indicators, number of expected classes, and class separation. Relatively small samples (*N* ≈ 100–200) might be adequate for relatively uncomplicated models with few indicators and highly differentiated classes [25].

Furthermore, recent simulation studies indicate that model performance depends on the joint contribution of sample size, number of indicators, and indicator quality. In a comprehensive Monte Carlo study, Wurpts and Geiser showed that LCA models with up to 12 binary indicators and two or three classes can be estimated with acceptable parameter recovery and convergence rates even with sample sizes around *N* = 200, particularly when indicators are theoretically grounded and of at least moderate quality [35]. Importantly, the authors demonstrated that increasing the number of indicators can partially compensate for smaller sample sizes, whereas models with very few indicators (< 5) are substantially more problematic. In light of these findings, the present sample size of 202 was considered acceptable for an exploratory LCA with 12 theoretically relevant indicators and a limited number of latent classes.

The eleven FA symptom criteria, along with impairment/distress, were used as categorical indicators in the models.

Once the most likely optimal solution had been identified, participants were assigned to the most likely class. Group comparisons on self-report variables were examined using Welch’s ANOVA, with Dunnett’s T3 post hoc tests where heteroscedasticity was found. Effect sizes were measured in terms of eta-squared (η²). These analyses were conducted to descriptively characterize differences in psychopathological, emotional, and metacognitive variables across the latent classes. Standardized z-scores were then entered into a set of univariate binary logistic regressions to test associations among membership in latent classes and clinical variables of interest (i.e., eating disorder psychopathology, depressive symptoms, metacognitive abilities, and emotion dysregulation), allowing us to identify which variables independently predicted class membership, accounting for shared variance among predictors. All statistical analyses were performed using IBM SPSS Statistics, Version 26.0. Statistical significance was defined at *p* < .05.

## Results

Table 1 presents the fit statistics for the one- to five-class solutions. Although the BIC favored the two-class solution, the three-class model showed a lower AIC, higher entropy relative to the two-class model, indicating clearer separation between profiles [34], and greater clinical interpretability. Given the exploratory nature of the study and the aim to identify clinically meaningful heterogeneity rather than the most parsimonious solution, the three-class model was retained.

**Table 1:** Absolute fit indices for measurement models

Model	LL	AIC	BIC	G^2	Entropy
1-class	– 1678.768	3425.537	3535.018	1776.176	–
2-class	–1456.531	3051.063	3279.333	1331.702	0.90
3-class	– 1395.899	2999.797	3343.857	1210.436	0.91
4-class	– 1365.545	3009.091	3468.94	1149.73	0.91
5-class	– 1338.048	3024.096	3599.735	1094.735	0.89

LL = log likelihood; AIC= Akaike Information Criteria; BIC= Bayesian Information Criteria; G^2 = Likelihood ratio/deviance statistic

**Fig. 1:**
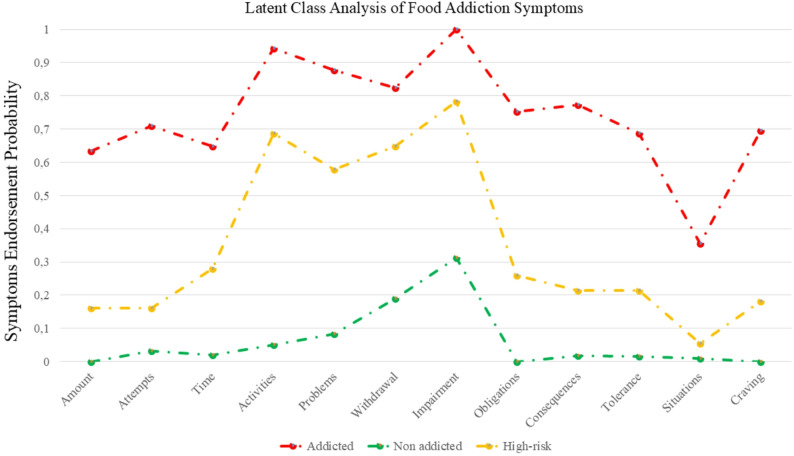
Profile plot from the latent class analysis of food addiction symptoms. The Y-axis shows, for each class, the average scores expressed as a proportion of the maximum possible value for each indicator. The X-axis lists the 11 food-addiction symptoms along with the impairment/distress criterion assessed by the YFAS 2.0

Specifically, we recognized three patterns (Fig. 1) by means of the item-response probability that we labeled as:

- Class 1: “addicted”, because, in this group, all the FA symptoms were highly endorsed and impairment criteria was present (*n* = 23, 11.4%);
- Class 2: “high-risk”, this group (*n* = 68, 33.7%) showed moderate endorsement of key functional symptoms, particularly impairment criteria, activities given up, withdrawal, and problems. At the same time, they reported low endorsement of behavioural excess symptoms, including tolerance, amount, time, attempts to cut down, consequences, situational cues, and craving. This pattern suggests a subgroup experiencing meaningful functional difficulties without the full range of addiction-like behavioural symptoms observed in the most severely affected class.
- Class 3: “non-addicted”, as members of this class had endorsed no FA symptoms (*n* = 111, 54.9%);

Table 2 describes the characteristics of the total sample and of each latent class. In the overall sample, most participants were single, had completed middle or high school, and no significant group differences emerged for age, BMI, civil status, or education.

**Table 2:** Characteristics of the total sample and by latent classes

		Total sample		Addicted	High-risk	Non- addicted			Post hoc
		*N* = 202		*n =* 23	*n =* 68	*n =* 111	χ^2^/F	*p*	
Age^a^		19.7	(7.2)	17.5 (2.6)	19.9 (7.2)	20.0 (7.7)	1.125	0.327	
Body mass index^a^		17.7	(3.0)	18.7 (2.5)	18.1 (2.9)	17.3 (3.0)	2.724	0.068	
Civil status^b^	Married	10	(5.0)	1 (4.3)	5 (7.4)	4 (3.6)	2.672	0.614	
	Single	187	(92.6)	22 (95.7)	62 (91.2)	103 (92.8)			
	Divorced	5	(2.5)	0 (0.0)	1 (1.5)	4 (6.6)			
Education^b^	Elementary	2	(1.0)	1 (4.3)	1 (1.5)	0 (0.0)	8.013	0.237	
	Middle school	121	(59.9)	17 (73.9)	40 (58.8)	64 (57.7)			
	High school	65	(32.3)	5 (21.7)	23 (33.8)	37 (33.3)			
	Master	14	(6.9)	0 (0.0)	4 (5.9)	10 (9.0)			

Table 3 presents the frequencies of AN subtypes across the three FA–related latent classes. The restrictive subtype (AN-R) was the most prevalent diagnosis across all classes, followed by atypical AN and the binge–purge subtype (AN-BP). Importantly, the distribution of AN subtypes did not differ significantly across the Addicted, High-risk, and Non-addicted classes (χ²(4) = 3.09, *p* = .544), indicating that FA-related symptom profiles were not associated with specific diagnostic subtypes.

**Table 3:** Frequencies of anorexia nervosa subtypes across the three food addiction–related latent classes

	Addicted		High-risk		Non addicted		Total
AN-*R*	12	(52.2)	46	(67.6)	77	(69.4)	135 (66.8)
AN-BP	5	(21.7)	8	(11.8)	12	(10.8)	25 (12.4)
AN-Atypical	6	(26.1)	14	(20.6)	22	(19.8)	42 (20.8)
Total	23	(100.0)	111	(25.0)	68	(100.0)	202 (100.0)

AN-R: Anorexia nervosa – restricting type: AN-BP: Anorexia nervosa – binge/purging type

Regarding psychopathological assessment, Table 4 reports the comparisons among the three latent classes across measures of EDE-Q, BDI-II, DERS, and MSAS. Significant group effects emerged for all EDE-Q subscales, with both the Addicted and High-risk groups scoring higher than the Non-addicted group. Depressive symptoms also differed significantly across groups, with the Addicted and High-risk classes reporting higher levels of depression compared to the Non-addicted group.

**Table 4:** Psychopathological differences among latent classes

		Addicted		High-risk		Non addicted							
		Mean	SD	Mean	SD	Mean		SD	F		*p*	η^2 a^	Post-hoc
EDE-Q	Restraint	3.5	2.1	4.1	1.8	2.7		2.1	10.520		< 0.001	0.10	2 > 3
	Eating concern	3.4	1.5	3.5	1.3	2.2		1.5	20.321		< 0.001	0.17	1,2 > 3
	Shape concern	4.6	1.5	4.8	1.3	3.5		2.0	13.455		< 0.001	0.12	1,2 > 3
	Weight concern	4.1	1.5	4.1	1.6	3.0		1.8	10.182		< 0.001	0.10	1,2 > 3
BDI-II		32.6	14.5	30.6	12.9	21.2		14.0	12.694		< 0.001	0.12	1,2 > 3
DERS	Non acceptance	21.1	7.7	18.7	6.0	15.0		7.1	8.945		0.001	0.10	1,2 > 3
	Goals	21.3	2.6	17.4	5.2	15.2		6.0	23.599		< 0.001	0.10	1 > 2,3
	Impulse	21.6	5.8	17.5	6.2	14.0		6.5	13.976		< 0.001	0.13	1 > 2,3
	Awareness	19.1	4.7	19.5	5.0	16.9		5.0	5.452		0.008	0.06	2 > 3
	Strategies	29.6	7.8	26.6	7.8	21.4		10.0	10.531		< 0.001	0.10	1,2 > 3
	Clarity	17.6	4.3	15.1	5.1	11.7		4.9	16.770		< 0.001	0.15	1,2 > 3
MSAS	Self-monitoring	14.1	4.9	16.4	4.0	19.2		4.9	11.585		< 0.001	0.13	1 < 2,3
	Differentiation/decentration	18.4	3.5	18.7	3.3	20.7		3.3	8.417		0.001	0.09	2 < 3
	Mastery	17.2	3.4	17.0	3.5	18.0		3.8	1.571		0.220		
	Others monitoring	10.1	2.5	10.6	2.2	11.1		2.8	1.402		0.257		

1: Addicted; 2: High-risk; 3: Non addicted

EDE-Q: Eating disorder examination – questionnaire; BDI-II: Beck depression inventory – II; DERS: Difficulties in emotion regulation scale; MSAS: Metacognition self-assessment scale

^a^ Only effect sizes of significant differences are displayed

For the DERS, the Addicted class showed greater difficulties than the Non-addicted group across non-acceptance, goals, impulse, strategies, and clarity subscales, while the High-risk class also scored higher than the Non-addicted group on most of these domains. A smaller effect was found for awareness, where only the High-risk group scored significantly higher than the Non-addicted group.

With regard to metacognitive functioning (MSAS), significant differences were observed for self-monitoring, with the Non-addicted group reporting higher abilities than both the Addicted and High-risk classes. A difference also emerged for differentiation/decentration, where the Non-addicted group scored higher than the High-risk group. No significant group differences were found for mastery and understanding others’ minds.

Partial η² values, reported only for variables showing a statistically significant ANOVA, indicated small-to-medium effect sizes (η² range = 0.06–0.17).

As shown in Table 5, binary logistic regression analyses were conducted to examine the associations between psychopathological and metacognitive variables with group membership (addicted, high-risk, non-addicted). The models accounted for a moderate proportion of variance (Nagelkerke’s R² = 0.281–0.381).

**Table 5:** Associations between group membership and psychopathological variables

		Addicted	High-risk	Non addicted
		OR (95% CI)	OR (95% CI)	OR (95% CI)
zEDE-Q	Restraint	0.360 (0.121-1.071)	1.035 (0.563-1.905)	1.363 (0.738-2.517)
	Eating concern	0.448 (0.096-2.098)	2.618 (1.211–5.662)*	0.410 (0.186-0.905)*
	Shape concern	4.981 (0.605-40.988)	1.615 (0.571-4.568)	0.542 (0.195-1.509)
	Weight concern	1.604 (0.361-7.118)	0.599 (0.261-1.373)	1.312 (0.571-3.017)
zBDI-II		0.552 (0.216 − 1.410)	962 (0.549-1.684)	1.197 (0.670-2.136)
zDERS	Non acceptance	1.021 (0.364-2.863)	1.007 (0.566-1.793)	0.861 (0.479-1.548)
	Goals	1.793 (0.619 − 5.190)	0.851 (0.494-1.464)	0.889 (0.513 − 1.540)
	Impulse	4.119 (1.198–14.163)*	1.234 (0.679-2.241)	0.602 (0.328-1.105)
	Awareness	1.069 (0.488-2.344)	1.619 (1.011–2.593)*	0.607 (0.369-0.999)*
	Strategies	0.525 (0.128 − 2.150)	1.180 (0.522 − 2.670)	1.121 (0.474-2.652)
	Clarity	1.453 (0.625-3.379)	1.138 (0.656-1.975)	0.744 (0.417-1.328)
zMSAS	Self-monitoring	0.589 (0.187-1.854)	1.737 (0.830-3.637)	0.613 (0.288-1.305)
	Differentiation/Decentration	0.493 (0.215 − 1.130)	0.566 (0.351-0.914)*	2.151 (1.280–3.615)**
	Mastery	1.643 (0.727-3.714)	1.268 (0.781-2.057)	0.672 (0.402-1.125)
	Others monitoring	1.134 (0.500-2.572)	0.972 (0.615-1.534)	0.989 (0.614-1.594)
Nagelkerke’s R^2^		0.359	0.281	0.381

*p < .05. **p < .01

z: Standardized scores; EDE-Q: Eating disorder examination - questionnaire; BDI-II: Beck depression inventory – II; DERS: Difficulties in emotion regulation scale; MSAS: Metacognition self-assessment scale; OR: odds ratio; CI: confidence interval

The addicted group was primarily characterized by difficulties with impulse control. Specifically, individuals in this group were significantly more likely to report problems regulating impulsive behaviors (OR = 4.12, 95% CI [1.20–14.16], *p* < .05). No significant associations were observed for eating disorder concerns, depressive symptoms, or metacognitive capacities.

The high-risk group showed a distinct profile, marked by elevated eating concerns (OR = 2.62, 95% CI [1.21–5.66], *p* < .05), greater difficulties with emotional awareness (OR = 1.62, 95% CI [1.01–2.59], *p* < .05), and poorer differentiation/decentration abilities (OR = 0.57, 95% CI [0.35–0.91], *p* < .05). These results suggest that high-risk individuals experience more eating-related preoccupations and emotion regulation difficulties, alongside weaker metacognitive skills in perspective-taking.

In contrast, the non-addicted group was characterized by comparatively lower levels of eating concerns (OR = 0.41, 95% CI [0.19–0.91], *p* < .05) and fewer problems with emotional awareness (OR = 0.61, 95% CI [0.37–0.999], *p* < .05). Moreover, this group demonstrated significantly better differentiation/decentration skills (OR = 2.15, 95% CI [1.28–3.62], *p* < .01), reflecting stronger metacognitive functioning.

Overall, the findings indicate that while the addicted group is distinguished by impulse dysregulation, the high-risk group exhibits a broader pattern of eating, emotional, and metacognitive vulnerabilities. Conversely, the non-addicted group is characterized by fewer eating-related and emotional difficulties and by stronger metacognitive resources.

## Discussion

This study represents, to our knowledge, the first attempt to disentangle the role of FA in patients with AN using an LCA approach. By adopting a person-centered approach based on FA symptomatology, we were able to identify three distinct profiles, namely addicted, high-risk, and non-addicted. Further, another strength of this work lies in the inclusion of an atypical AN subgroup, which has been largely overlooked in previous research but appears crucial for capturing the heterogeneity of FA across restrictive EDs.

## Food addiction as a transdiagnostic feature in AN

Our findings support the transdiagnostic nature of FA across AN spectrum. The frequencies of AN subtypes (restrictive, binge–purge, atypical) did not differ significantly across latent profiles, indicating that FA is not limited to those with binge-purge behaviors but may also emerge in restrictive and atypical forms of AN. This is consistent with prior evidence suggesting that addictive-like processes, such as compulsive preoccupation with food, craving, and loss of control, can manifest even in the absence of overt binge eating [26, 33, 34].

From another perspective, dietary restriction in patients with AN may be understood as a compensatory mechanism used by those with FA to balance episodes of excessive caloric intake. In fact, it is not uncommon to observe considerable variation in the food of individuals with AN: some eliminate all foods rich in fat and carbohydrates from their diet, while others may continue to eat small amounts of these foods while avoiding less “phobic” ones [36].

Another relevant point of discussion concerns the obsessive–compulsive features often observed both in AN and in addictive disorders. Recent large-scale evidence shows that obsessive-compulsive disorder (OCD) and obsessive-compulsive symptoms are associated with an increased risk of substance misuse, largely explained by shared genetic factors [37]. These shared obsessive characteristics may therefore represent an additional transdiagnostic vulnerability that contributes to the overlap between AN and FA. Thus, FA may represent a dimension that cuts across ED diagnoses, reflecting shared vulnerabilities in reward processing, impulsivity, and metacognition.

### Psychopathological burden in food-addicted groups

The findings demonstrate that patients in both the Addicted and High-risk profiles reported greater levels of eating disorder psychopathology, depression, and emotion dysregulation compared to their Non-addicted counterparts. Previous research also showed that FA exacerbates the severity of psychopathological symptoms in EDs [38]. Particularly, the elevated depressive symptoms and poor emotion regulation highlight the interplay between affective dysregulation and addictive eating tendencies. These findings underscore the clinical importance of screening for FA in AN, as its presence appears to mark a subgroup at risk of heightened psychological suffering.

Further, the result that the Addicted and High-risk profiles were characterized by greater eating disorder psychopathology raises the important question of whether associated elevations in depression, emotion dysregulation, and metacognitive impairment primarily reflect ED severity rather than FA-specific processes. Indeed, greater eating pathology is well known to co-occur with affective dysregulation and broader psychological distress. Accordingly, FA-related features in AN should not be interpreted as independent of core eating disorder severity, and it remains possible that some of the observed group differences are driven by more severe manifestations of the disorder rather than distinct addictive-like mechanisms.

At the same time, the differential psychological profiles observed across classes, particularly the distinct metacognitive and emotional characteristics of the High-risk group, suggest that FA symptom configurations may capture qualitative differences in vulnerability that are not fully reducible to a single severity continuum. From this perspective, FA symptoms may operate as markers of clinical complexity, identifying subgroups of individuals with AN who differ not only in severity, but in the configuration of affective and cognitive regulation processes. Future studies should incorporate assessment strategies specifically designed to disentangle FA-related processes from general ED severity, including multimethod and longitudinal approaches.

### The role of metacognitive abilities

The non-addicted group showed the most adaptive psychological profile, with fewer eating-related concerns, better emotional awareness, and significantly stronger differentiation/decentration abilities. These metacognitive resources appear to function as protective factors, allowing individuals to step back from intrusive food- or body-related cognitions and to adopt a non-egocentric perspective on themselves and others. In particular, good differentiation/decentration abilities are crucial for assuming a critical distance from one’s own body image, protecting from the distortion typical of these disorders and to prevent the immediate tendency to act. Such skills likely buffer against the escalation of addictive-like eating tendencies in AN and may facilitate more flexible coping strategies in the face of distressing emotions.

In contrast, the Addicted group was primarily characterized by impulsivity difficulties, highlighting how loss of control and poor self-regulation serve as central mechanisms underpinning compulsive, addiction-like eating behaviors. This pattern suggests that, in these patients, the addictive process may be less about distorted self-reflection and more about the immediate inability to modulate behavioral urges, echoing evidence linking FA to impulsive personality traits and deficits in inhibitory control.

The most complex and clinically relevant profile, however, emerged in the High-risk group. These individuals exhibited elevated eating concerns, deficits in emotional awareness, and impaired differentiation/decentration. This metacognitive vulnerability is particularly significant: poor differentiation hinders individuals’ ability to maintain critical distance from their own mental representations, increasing the likelihood of fusing thoughts with reality [39]. As a result, intrusive cognitions related to food, weight, and shape may be experienced as absolute truths, heightening preoccupation and rigidity. Moreover, difficulties in decentration compromise the ability to adopt another’s perspective, which can foster interpersonal misunderstandings, exacerbate social isolation, and reinforce self-focused rumination [40].

Taken together, this profile suggests that the High-risk group occupies an intermediate position: they do not yet display the full compulsive and impulsive pattern of the Addicted group, but their psychological vulnerabilities place them on a trajectory of significant clinical concern. The coexistence of heightened eating concerns, emotional dysregulation, and metacognitive impairments may render them particularly sensitive to stressors, increasing the probability of transitioning toward more severe and chronic addictive-like eating behaviors. Importantly, the dual role of differentiation/decentration, acting as a protective factor in the Non-addicted group but as a risk factor when impaired in the High-risk group, highlights its centrality as a mechanism of resilience versus vulnerability.

Importantly, the label “High-risk” should be interpreted with caution. In the present study, this designation is intended as a heuristic and clinically informed descriptor of a subgroup characterized by intermediate FA symptom expression and specific psychological vulnerabilities, particularly in metacognitive functioning and emotional awareness. However, given the cross-sectional design, we cannot determine whether individuals in this class are at increased risk of transitioning to more severe FA profiles or to poorer clinical outcomes. Therefore, the term “High-risk” should be considered provisional and hypothesis-generating rather than empirically validated. Longitudinal studies are needed to establish whether this profile is associated with adverse clinical trajectories over time.

Overall, these findings underscore that metacognitive dysfunctions are not peripheral features but lie at the core of vulnerability toward FA in AN. They also resonate with the growing literature that identifies metacognition as a transdiagnostic mechanism that shapes both symptom expression and clinical trajectories across eating and related disorders [24].

### Clinical implications

The identification of distinct FA-related profiles in AN has relevant clinical implications. Importantly, any consideration of FA-related features in AN must be situated within the primacy of nutritional rehabilitation and weight restoration, which remain the central treatment goals. FA-related symptom patterns are not intended to guide food avoidance or restriction, but rather to identify patients who may experience heightened distress, compulsivity, or loss of perceived control in relation to eating. These processes may be most relevant in later stages of treatment, once nutritional stabilization has been achieved, and may inform adjunctive therapeutic targets such as emotion regulation, cognitive flexibility, and metacognitive awareness, without conflicting with the need to increase and normalize food intake.

The clear association between impaired metacognitive capacities, especially differentiation/decentration sub-function, and FA symptomatology suggests that interventions targeting these functions may reduce vulnerability to addictive-like processes related to eating. In particular, Metacognitive Interpersonal Therapy (MIT), which aims to enhance self-monitoring, differentiation, and awareness of others’ mental states, may be especially well-suited for patients with AN presenting with FA-related features [23, 41]. By strengthening these abilities, clinicians may help patients distance themselves from rigid food- and weight-related cognitions, regulate emotions more adaptively, and decrease impulsive or compulsive patterns of behavior.

Another clinically relevant aspect concerns the well-documented diagnostic instability of EDs. It is well established that a substantial proportion of individuals with AN-R transition over time to binge–purge presentations or BN, with longitudinal studies reporting crossover rates ranging from one-third to more than half of AN patients [42, 43]. This trajectory is often associated with increasing symptom severity, greater impulsivity, and higher psychiatric comorbidity.

Importantly, the present study does not provide evidence that FA–related features predict diagnostic crossover, nor did FA-related profiles differ across AN subtypes in our sample. Nevertheless, when interpreted within a dimensional framework, FA-related symptom profiles may offer clinically meaningful information about patterns of psychological vulnerability that have been linked to diagnostic instability in prior longitudinal research. In particular, patients classified in the High-risk group were characterized by elevated eating concerns alongside selective impairments in metacognitive functioning, especially in differentiation and decentration capacities.

Such metacognitive difficulties may limit the ability to flexibly reflect on internal states, regulate emotional responses, and distance oneself from rigid food- and weight-related beliefs. In this sense, the High-risk profile may index a pattern of vulnerability associated with greater clinical complexity, rather than representing evidence of imminent transition to binge–purge phenotypes [42–46]. Conversely, stronger metacognitive capacities, as observed in the Non-addicted group, may function as protective factors against psychological processes associated with diagnostic instability. Longitudinal studies are needed to directly test these hypotheses.

## Limitations

Several limitations should be considered. First, the cross-sectional design does not permit inferences about the causal direction of relationships between metacognitive impairments, FA, and psychopathology. Longitudinal studies are needed to clarify whether poor differentiation/decentration is a predisposing factor for FA or a consequence of its persistence. Second, the use of self-report measures could have introduced social desirability or absence of introspective accuracy biases. Third, although the overall sample size was considered acceptable for exploratory LCA, the relatively small size of the Addicted class (*n* = 23) should be considered. Small class sizes may affect the stability and precision of parameter estimates, particularly in models with multiple indicators. Therefore, the present findings should be interpreted with caution, and replication in larger samples with more balanced class distributions is needed to confirm the robustness of the identified profiles. Moreover, the naturalistic recruitment strategy implies the inclusion of patients at different stages of the disorder and with varying levels of severity, which may have influenced the psychopathological presentation of AN. In addition, the relatively small proportion of individuals with the BP subtype represents a further limitation, particularly considering the focus on FA–related features, and may limit the generalizability of the findings to AN-BP presentations. Future studies including larger and more balanced AN subtype samples are warranted. Finally, biological or neurocognitive components were not included in the model and would further contribute to our understanding of FA in AN.

## Conclusions

In conclusion, this study advances the literature by providing the first person-centered exploration of FA in AN and by highlighting the critical role of metacognitive dysfunctions, particularly differentiation/decentration, in shaping vulnerability or resilience to FA. FA emerged as a transdiagnostic phenomenon across AN subtypes and was strongly linked with greater psychopathological burden. The differentiation between Addicted, High-risk, and Non-addicted AN groups may inform clinical decision-making and support more targeted interventions. Future research should further investigate the interplay between FA, metacognition, and affective dysregulation to refine therapeutic strategies and improve outcomes for individuals with AN.

## Data Availability

The datasets used and analyzed during the current study are available from the corresponding author upon reasonable request.

## Abbreviations

AIC: Akaike information criterion
AN: Anorexia nervosa
AN-BP: Anorexia nervosa–binge/purge type
AN-R: Anorexia nervosa–restricting type
BDI-II: Beck depression inventory–II
BED: Binge-eating disorder
BIC: Bayesian information criterion
BMI: body mass index
DERS: Difficulties in emotion regulation scale
DSM-5-TR: Diagnostic and statistical manual of mental disorders–5-text revision
EDE-Q: Eating disorder examination–questionnaire
EDs: Eating disorders
FA: Food addiction
LCA: Latent class analysis
MIT: Metacognitive interpersonal therapy
MSAS: Metacognition self-assessment scale
OR: Odds ratio
SCID-5-CV: Structured clinical interview for DSM-5–clinical version
YFAS 2.0: Yale food addiction scale 2.0
